# Improved Outcome of Severe Acute Pancreatitis in the Intensive Care Unit

**DOI:** 10.1155/2013/897107

**Published:** 2013-02-21

**Authors:** Polychronis Pavlidis, Siobhan Crichton, Joanna Lemmich Smith, David Morrison, Simon Atkinson, Duncan Wyncoll, Marlies Ostermann

**Affiliations:** ^1^Department of Critical Care, Guy's and St Thomas' NHS Foundation Trust, King's College London, London SE1 7EH, UK; ^2^Division of Health and Social Care Research, King's College London, London SE1 3QD, UK; ^3^Department of Abdominal Surgery, Guy's and St Thomas' NHS Foundation Trust, King's College London, London SE1 7EH, UK

## Abstract

*Background*. Severe acute pancreatitis (SAP) is associated with serious morbidity and mortality. Our objective was to describe the case mix, management, and outcome of patients with SAP receiving modern critical care in the Intensive Care Unit (ICU). *Methods*. Retrospective analysis of patients with SAP admitted to the ICU in a single tertiary care centre in the UK between January 2005 and December 2010. *Results*. Fifty SAP patients were admitted to ICU (62% male, mean age 51.7 (SD 14.8)). The most common aetiologies were alcohol (40%) and gallstones (30%). On admission to ICU, the median Acute Physiology and Chronic Health Evaluation (APACHE) II score was 17, the pancreatitis outcome prediction score was 8, and the median Computed Tomography Severity Index (CTSI) was 4. Forty patients (80%) tolerated enteral nutrition, and 46% received antibiotics for non-SAP reasons. Acute kidney injury was significantly more common among hospital nonsurvivors compared to survivors (100% versus 42%, *P* = 0.0001). ICU mortality and hospital mortality were 16% and 20%, respectively, and median lengths of stay in ICU and hospital were 13.5 and 30 days, respectively. Among hospital survivors, 27.5% developed diabetes mellitus and 5% needed long-term renal replacement therapy. *Conclusions*. The outcome of patients with SAP in ICU was better than previously reported but associated with a resource demanding hospital stay and long-term morbidity.

## 1. Introduction 

Acute pancreatitis affects 22.4 people per 100 000 of the general UK population per annum [1]. The incidence has risen by 46% over the last three decades with an epidemiological trend towards younger, female patients and alcohol as the main aetiology. Approximately 25% of patients with acute pancreatitis develop severe disease with associated organ dysfunction and require admission to the Intensive Care Unit (ICU) [2].

Although the mortality rate for the mild form of the disease is as low as 1%, severe acute pancreatitis (SAP) is still associated with high mortality and a prolonged stay in the ICU [3]. According to the Intensive Care National Audit & Research Centre (ICNARC), between 1995 and 2003 in the UK, 2677 patients with SAP were admitted to an ICU, and ICU mortality and hospital mortality were 31% and 42%, respectively [4].

There are several different scoring systems aimed at identifying patients with a high risk of a more complicated course. The Ranson and Glasgow (Imrie) criteria are the most commonly used [5, 6]. The Computed Tomography Severity Index (CTSI) is another score that has been shown to have good predictive value [7]. The Acute Physiology and Chronic Health Evaluation (APACHE) II and the Sequential Organ Failure Assessment (SOFA) scores are general severity of illness scoring systems that have also been shown to have good prognostic value in SAP [8, 9]. In 2007, Harrison et al. described the pancreatitis outcome prediction (POP) score which was derived from data in the ICNARC cohort and is based on arterial pH, age, serum urea, mean arterial pressure, pO_2_/FiO_2_ ratio, and total serum calcium (in order of decreasing impact) [10]. Although the original paper showed superiority over the aforementioned models, it has not yet been validated in other patient cohorts.

The objectives of our paper were to describe the case mix, current management, and outcome of patients with SAP in a large ICU in a tertiary care centre with a dedicated surgical pancreatitis team. In addition, we aimed to identify risk factors for mortality and to test the prognostic accuracy of commonly used scoring systems and the recently proposed POP score.

## 2. Materials and Methods

### 2.1. Setting

Guy's and St Thomas' NHS Foundation Trust is a tertiary referral centre for specialist services with 53 ICU beds and a dedicated surgical pancreatitis team.

### 2.2. Study Design

We retrospectively analysed available data between January 2005 and December 2010. In the absence of a consensus definition for SAP, we pragmatically included all patients with pancreatitis who were admitted to the ICU. Patients with chronic pancreatitis and patients who were transferred from other ICUs if their previous ICU stay was more than 48 hours were excluded. Clinical, laboratory, and imaging data were retrieved from the medical notes and electronic record system. We documented 6 conditions from the past medical history: history of pancreatitis, gallstones, diabetes mellitus, transplantation, chronic kidney disease, and liver cirrhosis. Detailed data regarding associated organ failure, need for organ support, type of nutrition, antibiotic use, complications, and radiological and surgical interventions throughout the whole stay in ICU were recorded. The criteria by the American-European Consensus Conference on ARDS were used to define Acute Lung Injury (ALI) and Acute Respiratory Distress Syndrome (ARDS) [11]. Acute Kidney Injury (AKI) was defined according to the Acute Kidney Injury Network criteria [12], and intra-abdominal hypertension (IAH) was defined as a persistently raised intravesical pressure >20 mmHg as per criteria agreed at an International Conference of Experts in 2006. In all patients, we recorded length of stay (LOS) in ICU and ICU and hospital outcome. In hospital survivors, we also documented the presence of diabetes and dialysis dependent end-stage renal failure. 

### 2.3. Scoring Systems and Definitions

We explored the predictive role of two general severity of illness scoring systems (APACHE II and SOFA scores) and two disease-specific scoring systems (POP score and Computed Tomography Severity Index (CTSI)). All scores were calculated using the worst values obtained in the first 24 hours after admission to ICU.

### 2.4. Statistics

Categorical variables were summarised using frequencies and proportions. Age was described as mean (standard deviation) and other continuous variables as median and interquartile range (IQR). Comparisons between subgroups were made using *t*- or Mann-Whitney tests for continuous variables, and the Chi-squared or Fisher's exact test, as appropriate, for categorical variables. The relationship between the number of computed tomography (CT) scans and CTSI was evaluated using Pearson's correlation. The correlation between APACHE II, SOFA, CTSI and POP scores and hospital outcome was assessed by receiver operating characteristic curve (ROC) analysis.

## 3. Results

### 3.1. Demographics

Between January 2005 and December 2010, 50 patients (31 male) were admitted to the ICU with SAP (Table 1). The mean age was 51.7 years (SD 14.8; range 16–85). Twenty patients (40%) had a previous episode of pancreatitis, and 8 patients (16%) were known to have gallstone disease, of whom 4 had previously undergone a cholecystectomy. The most common aetiologies of SAP were alcohol (40%) and gallstone disease (30%). On admission to the ICU, the median APACHE II score was 17 (IQR 12–19); median SOFA score, 5 (IQR 3–5); median POP score, 8 (IQR 5–12); and median CTSI 4 (IQR 2–7.5). 

### 3.2. Progress in ICU

During stay in ICU, 39 patients (78%) required respiratory support, 27 patients (54%) developed AKI of whom 22 (44%) received renal replacement therapy (RRT), and 31 patients (62%) needed treatment with vasoactive drugs (Table 1). The majority of patients (80%) tolerated enteral feeding via either a nasogastric tube (72%) or a nasojejunal tube (28%). Ten patients (20%) had to be converted to total parenteral nutrition (TPN). Patients on TPN had a longer median LOS in ICU (43 days versus 13 days, *P* = 0.0097) but there was no difference in ICU mortality (15% in patients on enteral nutrition compared to 30% in TPN group, *P* = 0.36).

There were no significant differences in APACHE II score on admission (*P* = 0.74) or length of stay in hospital (*P* = 0.23) between both groups. Forty-four patients were treated with antibiotics (empirical treatment for presumed sepsis (*n* = 23), confirmed sepsis (*n* = 21)). No patient received antibiotics prophylactically and no patient received treatment with octreotide.

**Figure 1:**
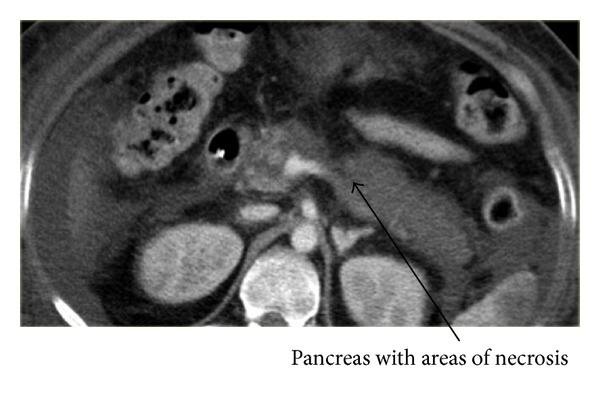
Representative CT scan of severe acute pancreatitis. CT scan with oral and intravenous contrast: >50% of the pancreas does not enhance consistent with necrotizing pancreatitis.

All patients had at least one CT scan of the pancreas with oral and intravenous contrast (Figure 1). The median number of CT scans per patient during stay in ICU was 2 (IQR 1–4) ranging from 1 to 14 scans per patient. Ten patients (20%) had evidence of IAH and 2 patients (4%) developed pseudoaneurysms (gastroduodenal and splenic artery). Twelve patients (24%) required CT-guided drain insertion for pancreatic cysts/collections, of whom 5 patients subsequently needed a surgical intervention. A total of 13 patients (26%) had surgery (necrosectomy (*n* = 5), laparotomy for abdominal compartment syndrome, bowel obstruction or small bowel perforation (*n* = 8)). The episode of bowel perforation occurred spontaneously and was not related to any intervention. Three patients (6%) underwent CT-guided embolisation of bleeding intraabdominal blood vessels following complications from pseudoaneurysms. 

### 3.3. Outcome

Median LOS in ICU and hospital was 13.5 days (IQR 6–30) and 30 days (IQR 16–70), respectively. ICU mortality and hospital mortality were 16% and 20%, respectively. Among hospital survivors, 11 patients (27.5%) developed insulin dependent diabetes mellitus and 2 patients (5%) needed long-term RRT for end-stage renal failure.

### 3.4. Risk Factor Assessment

There was no statistically significant difference in gender, age, past medical history, aetiology of SAP, or length of ICU and hospital stay between survivors and nonsurvivors. All non-survivors had evidence of AKI in contrast to 42% of survivors (*P* < 0.001).

**Figure 2:**
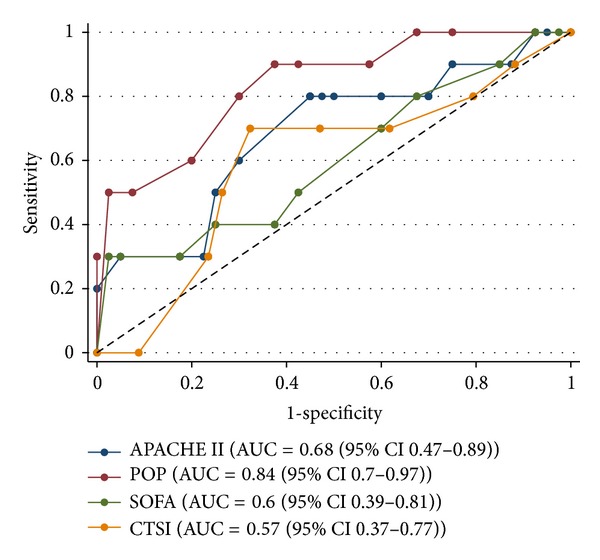
ROC curves for APACHE II, POP, SOFA, CTSI scores. ROC, receiver operator characteristic curve; AUC, area under curve; CI, confidence interval; APACHE, Acute Physiology and Chronic Health Evaluation; SOFA, sequential organ failure assessment; POP, pancreatitis outcome prediction; CTSI, Computed Tomography Severity Index.

Patients who died in hospital had a significantly higher POP score on admission compared to patients who died (Table 2). There was no significant difference in APACHE II, SOFA, and CTSI scores between hospital survivors and non-survivors. The POP score had the highest area under the receiver operating curve but the difference between POP and APACHE was not statistically significant ((area under the receiver operating characteristics curve 0.84 versus 0.68 (*P* = 0.25)) (Figure 2).

## 4. Discussion

This retrospective analysis provides a detailed description of the case mix, management, and outcome of patients with SAP receiving modern critical care in a tertiary care centre in the UK. High morbidity with organ failure, multiple imaging requirements, invasive procedures, and long-term complications after discharge (diabetes and end-stage renal failure) are still the characteristics of the disease. In particular, the development of AKI is associated with a high risk of dying, as previously described [13].

The mortality rates described in this study are much lower than our group and others have previously reported [4, 14]. The likely explanation is due to differences in the patient cohorts and variations in clinical management, including criteria for admitting patients to the ICU. We note that in our own case series from the 1990s the median APACHE II score on admission to ICU was 18 compared to 17 in our more recent analysis, and the ICU mortality was 39%. Changes in medical management may have also contributed, including a less invasive approach as advocated over the last 7 years with focus on fluid resuscitation, early imaging, rational antibiotic use, use of enteral nutrition, and minimally invasive surgical procedures [15, 16]. 

Nutritional support has been the focus of numerous studies. Meta-analyses have suggested improved outcomes with enteral compared with parenteral nutrition [17]. The largest meta-analysis included a total of 27 randomized controlled trials [18]. Combined analysis of seven trials comparing enteral to parenteral nutrition showed a significant reduction in infectious morbidity and hospital length of stay (202 patients, mean difference 4 days between groups), and a trend towards reduced organ failure. Other benefits were seen in individual trials of enteral nutrition including a reduction in oxidative stress and hastening of resolution of the disease process. The majority of patients in our cohort tolerated enteral nutrition. This is a change from our own previous case series when only 25% of patients were fed enterally and a large proportion (33%) did not receive any nutritional support and were kept nil by mouth for >5 days [14].

Infection is a feared complication in patients with SAP. Recommendations on the role of antibiotics are conflicting [19, 20]. One systematic review concluded that prophylactic antibiotics decreased mortality in severe pancreatitis, but not the rate of infected pancreatic necrosis [19]. In contrast, a subsequent meta-analysis of seven trials detected no mortality benefit or reduction in the incidence of infected necrosis [20]. In our practice, broad spectrum antibiotics are not routinely administered to patients with SAP. However, the majority of patients received antibiotics during their stay in ICU empirically or for confirmed infections. 

Fifty percent of our patients needed a surgical and/or radiological intervention. In the case of infected necrosis, our first line treatment was a minimally invasive approach as advocated by the literature [21]. Surgical debridement was reserved for patients in whom the minimally invasive methods failed to resolve the fluid collections or if the collections were not accessible by these methods. Of significance was the number of CT scans required per patient while in ICU, that is, a median number of 2 scans per patient ranging from 1 to 14. The associated high radiation exposure has been highlighted in previous studies [22]. The low CTSI scores in our cohort may be attributed to early patient scanning.

Several scoring systems are in use to estimate the prognosis of patients with SAP [5–10]. In the original study, the POP score was superior to other methods. In our cohort, we did not detect a significant difference between the APACHE II and POP scores. However, an adequately powered validation study is necessary to evaluate the role of the POP score in more detail. 

It is important to acknowledge potential limitations of this analysis. The obvious weakness is the size of the cohort and the single centre setting. As a result, we cannot exclude any confounding influences specific to our clinical practice. We also acknowledge that univariate analysis of a relatively small sample needs to be interpreted with caution. Ideally we would have liked to perform a multivariate regression analysis but our sample size and number of events were too small. Finally, we were unable to calculate the Ransom and Glasgow Scores retrospectively due to missing values.

## 5. Conclusions 

This paper describes improved ICU and hospital outcomes in a cohort of patients with SAP receiving modern critical care in a tertiary care centre at the cost of a prolonged, resource demanding stay in hospital and significant morbidity. Larger studies will be required to verify the presented findings, evaluate morbidity as well as quality of life after discharge from hospital, and, if possible, estimate the associated healthcare costs.

## Figures and Tables

**Table 1:** Baseline characteristics and outcomes.

Parameter	Prevalence (*n* = 50)
Male gender (%)	31 (62%)
Mean age (SD; range)	51.7 (14.8; 16–85)
Past Medical History	
Pancreatitis	40%
Diabetes mellitus	24%
Gallstone disease	16%
Liver cirrhosis	8%
Chronic kidney disease	8%
Transplantation	4%
Aetiology of severe pancreatitis	
Alcohol	40%
Gallstone disease	30%
Drug induced	6%
Hypocalcaemia	4%
Post ERCP	2%
Hypertriglyceridemia	2%
Idiopathic	16%
Transfer from other hospital	48%
Severity of illness on admission to ICU	
APACHE II score, median (IQR)	17 (12–19)
SOFA, median (IQR)	5 (3–8)
POP, median (IQR)	8 (5–12)
CTSI, median (IQR)	4 (2–7.5)
Associated organ failure	
AKI	54%
ALI	56%
IAH	20%
Need for respiratory support	78%
Need for RRT	44%
Treatment with vasoactive drugs	62%
Nutrition	
TPN	20%
Enteral nutrition only	80%
Interventional treatment	
Drain insertion	24%
Surgical intervention	26%
Embolisation	7.5%
Outcome	
ICU mortality	16%
Hospital mortality	20%
LOS in ICU, median (IQR)	13.5 (6–30)
LOS in Hospital, median (IQR)	30 (16–70)
Diabetes mellitus in hospital survivors	11 of 40 survivors
End stage renal failure in hospital survivors	2 of 40 survivors

ICU: intensive care unit; ERCP: Endoscopic Retrograde Cholangiopancreatography; SD: standard deviation; IQR: interquartile range; APACHE: Acute Physiology and Chronic Health Evaluation; SOFA: sequential organ failure assessment; POP: pancreatitis outcome prediction; CTSI: Computed Tomography Severity Index; AKI: acute kidney injury; ALI: acute lung injury; IAH: intraabdominal hypertension; RRT: renal replacement therapy; TPN: total parenteral nutrition; LOS: length of stay.

**Table 2:** Average scores of patients who survived and those who died in hospital.

Score	Hospital survivors	Hospital nonsurvivors	*P *
APACHE II	14.5 (11–18.5)	18.5 (17–22)	0.080
POP	7 (4.5–11)	13 (11–19)	0.001
SOFA	5 (3–7.5)	5.5 (4–13)	0.323
CTSI	3 (2–7)	5.5 (2–8)	0.508

All values are given as median (interquartile range).

ROC: receiver operator characteristic curve; AUC: area under curve; CI: confidence interval; APACHE: Acute Physiology and Chronic Health Evaluation; SOFA: sequential organ failure assessment; POP: pancreatitis outcome prediction; CTSI: Computed Tomography Severity Index.
